# Pre- and post-discharge physical activity in acute myocardial infarction: Relation to clinical events across 5-year follow-up

**DOI:** 10.1016/j.ajpc.2026.101641

**Published:** 2026-04-17

**Authors:** Rik Dijkman, Sophie H. Kroesen, Bram M.A. van Bakel, Robert Jan M. van Geuns, Aukelien C. Dimitriu-Leen, Thijs M.H. Eijsvogels, Dick H.J. Thijssen

**Affiliations:** a Department of Medical BioSciences, Radboud University Medical Center, Geert Grooteplein Zuid 10, Nijmegen, GA 6525, the Netherlands; b Department of Cardiology, Radboud University Medical Center, Geert Grooteplein Zuid 10, Nijmegen, GA 6525, the Netherlands; c Research Institute for Sports and Exercise Sciences, Liverpool John Moores University, Tom Reilly Building, Byrom Street L3 3AF, Liverpool, United Kingdom

## 1 Introduction

Coronary artery disease (CAD) remains a leading cause of morbidity and mortality worldwide [1]. Increasing habitual physical activity (PA) is recommended in contemporary guidelines as it attenuates disease progression and improves clinical outcomes. However, exercise-based cardiac rehabilitation (CR) typically commences weeks to months after myocardial infarction (MI), leaving the immediate post-discharge period poorly defined regarding PA and its safety. Therefore, we examined the association between objectively measured post-discharge PA and short-term major adverse cardiovascular events (MACE) at 1-, 3-, and 6-months follow-up in patients recovering from MI. In addition, we explored the relationship between pre-admission PA and post-discharge PA, and assessed their associations with long-term (5-year) MACE.

## 2 Methods

### 2.1 Study population and design

This prospective cohort study included patients hospitalised for MI at the Radboud university medical center between January 2019 and September 2021. Eligible patients were ≥18 years of age and diagnosed with either ST-Elevation MI (STEMI) or Non-ST-Elevation MI (NSTEMI). Patients with severe mobility limitations or acute heart failure at admission were excluded. The study was approved by the ethics committee of the Radboud university medical center, Nijmegen (#2018–4537).

### 2.2 Data collection

Post-discharge PA was objectively assessed using a thigh-worn validated accelerometer (ActivPAL3 micro; PAL Technologies Ltd, Glasgow, UK), during the first week after discharge. Measured activities with a metabolic equivalent of task (MET) score between 1.5–3, ≥3, and ≥6 were categorised as light-intensity physical activity (LIPA), moderate-vigorous physical activity (MVPA), and VPA, respectively. Pre-admission PA was assessed retrospectively using the validated Short Questionnaire to Assess Health-enhancing Physical Activity (SQUASH), and subsequently used to calculate MET scores. In this study, leisure-time PA was used as an indicator of patients’ pre-admission PA level. MACE were recorded during 5-year follow-up and defined as a composite of all-cause mortality, reinfarction, unplanned revascularisation (PCI or CABG), acute heart failure, and stroke. Clinical data were identified through electronic medical records.

### 2.3 Statistical analysis

Patients were stratified into high and low post-discharge PA groups, based on the median value. Group differences were assessed using appropriate parametric or non-parametric tests. Modified Poisson regression models with robust standard errors were used to estimate relative risks (RRs) and corresponding 95% confidence intervals (CIs) for the association between post-discharge PA and the occurrence of MACE at 3- and 6-months. Sequential models were constructed, adjusted for pre-admission PA, length of stay, and peak high-sensitive cardiac Troponin-T (hs-cTnT). Kaplan-Meier (KM) survival curves were constructed to compare long-term time-to-MACE by both pre-admission PA and post-discharge PA groups, with differences between high *versus* low PA groups assessed by the log-rank test. Subsequently, Cox proportional hazards regression was used to estimate hazard ratios (HRs) and corresponding 95% CIs for the 5-year follow-up period. Sequential models were constructed, adjusted for age and sex, the alternate PA domain (*i.e.*, pre-admission or post-discharge), peak hs-cTnT, and length of stay. All analyses were performed in Python 3.12 (Python Software Foundation, Wilmington, DE, USA) using the *statsmodels, lifelines*, and *scipy* packages.

## 3 Results

Of 200 enrolled patients, 165 (83%) had accelerometer data of sufficient quality and clinical follow-up data to be included in the analyses. Mean age was 65 ± 10 years, 50% presented with STEMI, 78% underwent PCI, and 9% CABG. Median follow-up was 4.9 years, during which 50 patients experienced MACE (74 total MACEs), most commonly revascularisation (*n* = 28), reinfarction (*n* = 18), and mortality (*n* = 16). Median post-discharge PA was 3.4 h/day, including 24 min/day of MVPA. Baseline characteristics were comparable between high and low PA groups, except for longer length of stay and more CABG in the low PA group.

**Fig. 1:**
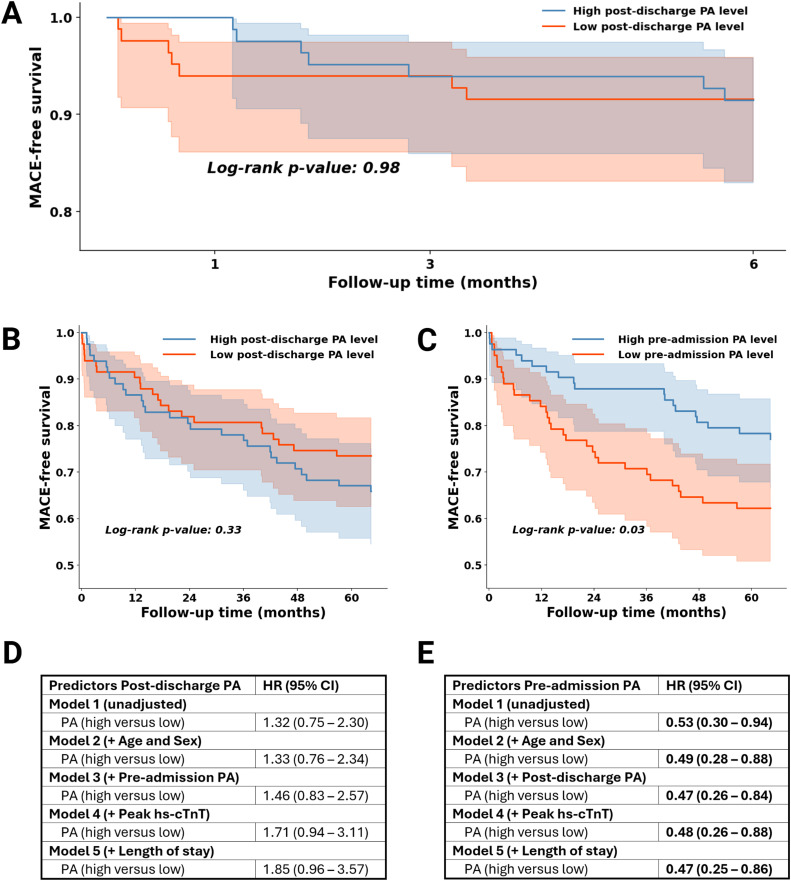
(**A**) Kaplan-Meier survival curve for high post-discharge PA (*N* = 82, blue) and low post-discharge PA (*N* = 83, orange) related to occurrence of MACE across 6 month follow-up. (**B**) Kaplan-Meier survival curve for high post-discharge PA (*N* = 82, blue) and low post-discharge PA (*N* = 83, orange) related to occurrence of MACE across 5 year follow-up. (**C**) Kaplan-Meier survival curve for high pre-admission PA (*N* = 83, blue) and low pre-admission PA (*N* = 82, orange) related to occurrence of MACE across 5 year follow-up. (**D**) Sequentially adjusted Cox proportional hazards regression for post-discharge PA and 5-year follow-up. (**E**) Sequentially adjusted Cox proportional hazards regression for pre-admission PA and 5-year follow-up. Bold values indicate significant difference (*p* < 0.05).Abbreviations: MACE, Major Adverse Cardiovascular Events; PA, Physical Activity; hs-cTnT, high-sensitive cardiac Troponin-T; HR, Hazard Ratio; 95% CI, 95% Confidence Interval.

At 1 month, 5 patients experienced MACE in the low post-discharge PA group *versus* 0 patients in the high post-discharge PA group (Fig. 1, Panel A). At 3- and 6-months, both high and low post-discharge PA groups had 5 and 7 patients with MACE, yielding comparable absolute risks (6.1% *versus* 6.0% at 3-months; 8.5% *versus* 8.4% at 6-months) (Fig. 1, Panel A). Poisson regression analyses showed no significant differences in RR for MACE at 3- and 6-months between high and low post-discharge PA groups, and results were unaltered after adjustment for confounders. Pre-admission and post-discharge PA were not associated. Across 5-year follow-up, post-discharge PA was not associated with MACE (Fig. 1, Panel B). In contrast, higher pre-admission PA was associated with fewer events and lower long-term MACE risk (adjusted HR 0.47 (95% CI: 0.25–0.86)) (Fig. 1, Panel C and E), whereas post-discharge PA was not (adjusted HR 1.85 (95% CI: 0.96–3.57)) (Fig. 1, Panel D).

## 4 Discussion

Although PA levels increase rapidly after discharge following MI, evidence-based guidance on the safety of early post-discharge PA is lacking [2]. In this study, post-discharge PA was not associated with short-term MACE or long-term outcomes. To our knowledge, this is the first study to relate objectively measured PA in the first week after discharge to clinical outcomes. These findings are clinically relevant given the trend towards shorter post-MI length of hospital stays and the substantial increase in PA after discharge [3]. Importantly, results remained unchanged after adjustment for clinical factors, including treatment strategy, length of stay, peak hs-cTnT, and in sensitivity analyses for VPA. Overall, these findings indicate that early mobilisation after MI is common and appears safe, supporting promotion of early PA in this population.

Contrary to our hypothesis, post-discharge PA was not associated with pre-admission PA, suggesting that early post-discharge behaviour does not reflect habitual activity patterns. This discrepancy may reflect differences in data collection (questionnaires *versus* objective assessment), but may also relate to PA context. More specifically, pre-admission PA represents lifestyle behaviour, while post-discharge PA reflects short-term adaptation following a major clinical event. In contrast, higher pre-admission PA was associated with improved 5-year MACE-free survival, consistent with previous evidence on long-term protective effects of habitual PA [4], further supporting the importance of an active lifestyle prior to MI. Together, these findings indicate that pre-admission and post-discharge PA represent distinct behavioural constructs, and that patients do not necessarily resume prior activity levels after MI. Clinically, the early post-discharge phase may represent a ‘teachable moment’ to support behaviour change [5], underscoring the need for early guidance and structured support, regardless of pre-MI activity levels.

This study has some limitations. First, the sample size has limited power to detect small differences in MACE risk, given the low absolute risk of exercise-induced events [6]. Second, the observational design precludes causal inference and residual confounding cannot be excluded. Third, post-discharge PA was assessed only during the first week after discharge, without repeated measurements during follow-up, limiting insight into longer-term activity patterns. Finally, information on post-discharge management, including participation in CR, was not available. Although our findings suggest that early post-discharge PA appears safe, they should be interpreted as hypothesis-generating.

## 5 Conclusion

Post-discharge PA during the first week after MI was not associated with short-term MACE risk, whereas higher pre-admission PA was associated with improved 5-year MACE-free survival. Post-discharge PA did not correlate with pre-admission PA, indicating that early activity does not necessarily reflect habitual behaviour. Future randomized controlled trials with standardized exercise interventions and longitudinal PA monitoring are needed to confirm our hypothesis-generating observations and determine whether early post-discharge PA causally influences clinical outcomes and to inform optimisation of early CR and mobilisation.

## Funding

This study was partly supported by the Dutch Heart Foundation (senior E-Dekker grant #2017T051) and by Health∼Holland (#R0006698).

## Author agreement

The authors have agreed to the submission of this manuscript “Pre- and post-discharge physical activity in acute myocardial infarction: Relation to clinical events across 5-year follow-up″ and the materials in this manuscript have not been previously published nor are in consideration for publication elsewhere.

## CRediT authorship contribution statement

**Rik Dijkman:** Writing – review & editing, Writing – original draft, Visualization, Validation, Methodology, Investigation, Formal analysis, Data curation, Conceptualization. **Sophie H. Kroesen:** Writing – review & editing, Visualization, Methodology, Investigation, Formal analysis, Conceptualization. **Bram M.A. van Bakel:** Writing – review & editing, Methodology, Investigation, Data curation, Conceptualization. **Robert Jan M. van Geuns:** Writing – review & editing, Supervision, Funding acquisition. **Aukelien C. Dimitriu-Leen:** Writing – review & editing, Supervision, Data curation. **Thijs M.H. Eijsvogels:** Writing – review & editing, Supervision, Methodology, Funding acquisition, Formal analysis, Conceptualization. **Dick H.J. Thijssen:** Writing – review & editing, Supervision, Methodology, Funding acquisition, Formal analysis, Conceptualization.

## Declaration of competing interest

The authors declare that they have no known competing financial interests or personal relationships that could have appeared to influence the work reported in this paper.

## Data Availability

Data is available upon reasonable request via the corresponding author.
